# Methamphetamine-Induced Cardiomyopathy (MACM) in a Middle-Aged Man; a Case Report

**Published:** 2018-01-20

**Authors:** Zulfiqar Qutrio Baloch, Muhammad Hussain, Shabber Agha Abbas, Jorge L. Perez, Muhammad Ayyaz

**Affiliations:** 1 Department of Internal Medicine, Brandon Regional Hospital, Brandon, Florida, USA.; 2 R-Endocrinology, Hamilton, New Jersey, USA.

**Keywords:** Methamphetamine, Cardiomyopathies, Emergency Service, Hospital

## Abstract

The development of methamphetamine-associated cardiomyopathy (MACM) represents a severe complication of chronic methamphetamine abuse. MACM-induced irreversible structural and functional changes in the heart can eventually lead to decompensated heart failure, ultimately requiring heart transplantation. In this case report we present a 47-year old male with a previous history of chronic amphetamine abuse who presented to the emergency room with severe dyspnea at rest associated with mild substernal non-radiating chest pain. He denied any previous cardiac history but had a positive urinary toxicology for methamphetamine. A complete cardiac workup ruled out all other etiologies. The patient required a 3-week intensive pharmacotherapy intervention to stabilize acute heart failure symptoms. At discharge he was classified as having New York Association Class III (NYHA-III) heart failure. His medical symptoms did not improve and he was considered for heart transplantation. With the increase in availability and abuse of methamphetamine, case of MACM such as ours are more frequently being encountered in the emergency departments. In addition to raising awareness, our case provides an outline of how MACM patients likely may present and the subsequent morbid sequela. Clinicians should maintain a high degree of suspicion when assessing all patients with a history of methamphetamine abuse. Early cardiac evaluation can help identify ventricular compromise in asymptomatic patients providing an opportunity to intervene prior to the development of irreversible MACM.

## Introduction:

Methamphetamine is a highly addictive central nervous system (CNS) stimulant produced from the parent compound amphetamine, which is frequently used to treat attention deficit hyperactivity disorder. Methamphetamine and related compounds are frequently abused as recreational drugs, and are the second most widely used illicit drug in the United States after cannabis (1). Apart from CNS stimulation, methamphetamine has significant adverse effects on the peripheral nervous system associated with both short-term and long-term usage. The primary mechanism of action of methamphetamine is the increased release and decreased uptake of catecholamines at the neuronal synapse producing a marked effect on the cardiovascular system. Common physical symptoms after acute intoxication are chest pain, hypertension, arrhythmias, myocardial infarction, and palpitations (2). Chronic methamphetamine abuse can lead to development of severe cardiovascular complications such as coronary artery disease, acute myocardial infarction, ischemic cardiomyopathy, methamphetamine-associated cardiomyopathy (MACM), aortic dissection, malignant hypertension, dysrhythmias, and sudden cardiac death (3, 4). We discuss the case of a patient with dyspnea at rest due to severe cardiomyopathy following three years of methamphetamine use.

## Case presentation:

A 47-year-old male presented to the emergency department with a chief complaint of severe dyspnea at rest associated with mild substernal non-radiating chest pain. He denied palpitations, productive cough, abnormal sound while breathing, difficulty swallowing, history of a cardiac problem, recent viral illness, recent travel history, or any recent exposure to anyone having a similar presentation. His past medical and surgical histories were not significant. Family history was not significant for cardiomyopathy. Upon urinary drug screening his test was positive for methamphetamine use. He stated that he had been using methamphetamine for the past three years. He denied use of any other recreational drugs and denied excessive alcohol use. 

**Figure 1:**
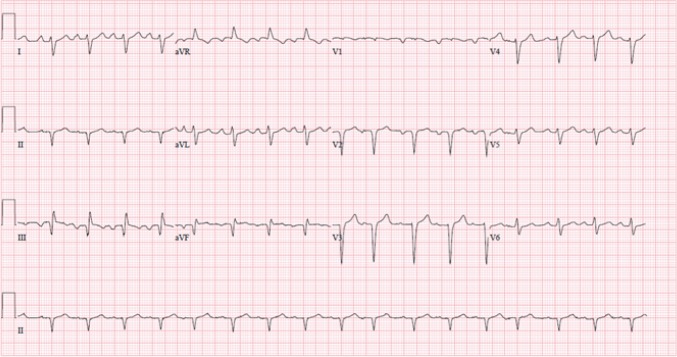
12 leads electrocardiography of the patient

**Figure 2:**
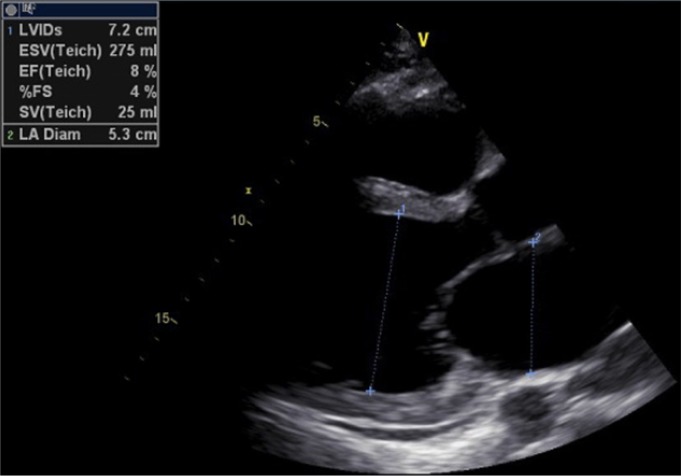
Transthoracic echocardiography of the patient (parasternal long axis view)

On physical examination he was found to be alert and oriented to time, place, and person. Vital signs on presentation were: blood pressure: 90/70 mmHg, pulse rate: 120 beats/minute, and respiratory rate: 26 breaths/minute. Arterial oxygen saturation was 88% in room air. Examination of the chest revealed bilateral diffuse crackles, most pronounced on lung bases. A chest radiograph showed pulmonary congestion and cardiomegaly. An electrocardiogram (ECG) showed sinus tachycardia, low voltage, poor progression of R-wave in precordial leads, incomplete LBBB, extreme right axis deviation with RV strain, S1 Q3 inverse T3 pattern, Q-wave in 2-3 AVF suggestive of old MI (Figure 1). Cardiac troponin testing was within reference range. To further narrow down the differential diagnosis a complete work-up was ordered including: complete blood count (CBC), basic metabolic profile (BMP), lipid profile, thyroid function tests, creatinine phosphokinase, erythrocyte sedimentation rate (ESR), blood morphology, liver function test (LFT), protein level, and iron studies. These were all found to be within reference range. Moreover, immunological assays [immunoglobulin M (IgM) and immunoglobulin G (IgG) anti-Epstein–Barr virus] and toxoplasmosis serology were found to be negative. Fasting transferrin saturation test was normal. Transthoracic echocardiography (TTE) revealed severe left ventricular (LV) and left atrial (LA) dilatation, extremely impaired systolic function with left ventricular ejection fraction (LVEF) below 15% (actual was 8%), full size RV, normal mitral and aortic valve, normal aortic root size (Figure 2). Subsequent coronary angiography testing found no coronary artery disease. 

Treatment for acute heart failure exacerbation was immediately initiated including use of lisinopril, carvedilol, and furosemide. Following three weeks of intensive pharmacotherapy and considerable clinical improvement the patient was discharged from the hospital. At time of discharge the patient was classified as having New York Heart Association Class III (NYHA-III) heart failure. Outpatient follow-up visits were scheduled at 1, 2, and 3 months, respectively. No clinical or echocardiographic improvements were noted during all follow-up visits. After optimal medical treatment the patient’s symptoms were not improved and he was considered for heart transplantation. 

## Discussion:

The incidence of methamphetamine abuse among young population has increased with predominance among college students in the United States (5). There are several reasons behind this increased frequency. One explanation is the relatively easy availability and access through the Internet as depicted in the World Drug Report 2016 (1). Another reason is the inexpensive pricing making it a suitable drug of choice even for those with economic constraints. As an agent having a long duration of action it holds a particular appeal for those desiring chronic stimulant effects (1). 

Methamphetamine can be ingested orally, smoked, snorted, or injected. Smoking or injecting the drug produces an immediate effect and makes the patient prone to developing adverse health problems, while raising the addiction potential. Methamphetamine has good lipid solubility compared to its parent compound, amphetamine, and can more readily cross the blood brain barrier. The drug has a marked effect on both central and peripheral nervous systems. By facilitating increased release and decreased reuptake of catecholamines from nerve terminals, it stimulates alpha- and beta-adrenergic receptors. The pronounced effects of the drug are on multiple organ systems including the cardiovascular system leading to hypertension and tachycardia in the short-term and severe cardiovascular complications such as myocardial infarction, dysrhythmias, ventricular hypertrophy, pulmonary edema, hypertension, cerebral stroke, cerebral hemorrhage, seizures, psychosis, and in certain cases even death. Acute coronary syndrome can also be a result of cardiac damage as evident by published case reports on acute coronary syndrome and coronary artery rupture following drug abuse (6, 7). The prevalence of methamphetamine-induced heart failure is also considerable, with at least one study reporting an up to 5% incidence rate in patients presenting to the emergency department with heart failure (8). A recent article pointed out that the experience of emergency health care personnel, especially mental health professionals, in dealing with patients presenting with acute methamphetamine abuse is quite complex. They have to employ a range of different strategies to stabilize patients who are acutely engaged in hyperactive and stimulant behavior (9).

The development of cardiomyopathy is a severe complication of chronic methamphetamine abuse and is rarely a sign of acute abuse. In a study on 107 young patients presenting with idiopathic cardiomyopathy, 40% of patients were found to have chronically abused methamphetamines in subsequent interviews and urinalyses (3). Our patient presented with severe dyspnea at rest and on further investigation was found to have severe systolic dysfunction due to cardiomyopathy following three years of chronic methamphetamine abuse. Patients fitting this profile develop structural and functional changes in myocytes such as atrophy, hypertrophy, eosinophilic degeneration, and fibrosis (10). Histopathological assessment is likely to reveal cardiomyopathic lesions including slight interstitial fibrosis. These changes eventually lead to decompensated heart failure that requires heart transplantation for continued survival (8). Certain patients who achieve symptomatic relief with pharmacotherapy alone may benefit from biventricular cardiac pacing or automatic implantable cardiac defibrillators (4). The decision to pursue such interventions depends on many factors such as treatment compliance, likelihood of relapse, presence of co-morbidities, and risk of device infection, especially in intravenous drug abusers (4).

Schurer and colleagues recently investigated the histopathological impact of methamphetamine discontinuation in chronic abuse patients by following the clinical characteristics, histopathological features, and clinical outcome in a cohort of 30 patients presenting with MACM (10). Patients with LVEF <40%, whose endomyocardial biopsy confirmed MACM were followed for mean 35 ± 22 months post-study inclusion. They compared histopathology changes between those who discontinued the drug and those who continued. Patients enrolled in the study were highly symptomatic and 83.3% of them had New York Heart Association functional class III or IV dyspnea. The mean duration of methamphetamine abuse was 5.7 years. Baseline LVEF was 19 ± 6%. At follow-up, 23 patients had stopped taking methamphetamine whereas 7 continued. Those who continued had persistently, severely impaired LVEF and LV dilatation; whereas those who discontinued had LV remodeling and improved LVEF. Discontinuation was also associated with improved symptoms. In terms of the primary endpoint of composite death, nonfatal stroke, and rehospitalization for heart failure, these events occurred more frequently in the group that continued abusing methamphetamine (p=0.037). With respect to histopathologic changes, only fibrosis was found to be associated with the duration of methamphetamine abuse, occurring in a more severe form with longer abuse duration. No difference in inflammatory changes was observed between the 2 groups. 

Our patient had an LVEF of 8%, which is typical as patients with MACM have a significantly lower LVEF compared to patients with cardiomyopathy from all other causes (3). In addition to that, our patient had no significant risk factors responsible for causing cardiomyopathy and heart failure, except for methamphetamine abuse. Patients with a modestly reduced LVEF may demonstrate a more severe ventricular dilatation than those with other causes of cardiomyopathy on imaging. MACM and its clinical sequela warrant attention, especially in an era of increasing use of methamphetamines and related compounds. Recognizing MACM and associated heart failure in the emergency setting is essential for initiating both short-term and long-term treatments, which hold implications for survival. 

## Conclusion:

Methamphetamine-induced cardiomyopathy or MACM and its clinical sequela warrant attention, especially in an era of increasing use of methamphetamines and related compounds. Clinicians should maintain a high degree of suspicion when assessing all patients with a history of methamphetamine abuse, especially chronic users and those who may have preliminary signs or symptoms of cardiovascular compromise. In patients presenting with idiopathic cardiomyopathy, methamphetamine abuse should be in the differential diagnosis if the patient’s profile is suspicious. Early cardiac evaluation can identify ventricular compromise in asymptomatic patients prior to the development of MACM and should be considered for screening purposes.

## References

[1] World Drug Report 2016 http://www.unodc.org/doc/wdr2016/WORLD_DRUG_REPORT_2016_web.pdf.

[2] Lan K-C, Lin Y-F, Yu F-C, Lin C-S, Chu P (1998). Clinical manifestations and prognostic features of acute methamphetamine intoxication. Journal of the Formosan Medical Association= Taiwan yi zhi.

[3] Yeo K-K, Wijetunga M, Ito H, Efird JT, Tay K, Seto TB (2007). The association of methamphetamine use and cardiomyopathy in young patients. The American journal of medicine.

[4] Paratz ED, Cunningham NJ, MacIsaac AI (2016). The cardiac complications of methamphetamines. Heart, Lung and Circulation.

[5] McCabe SE, Knight JR, Teter CJ, Wechsler H (2005). Non‐medical use of prescription stimulants among US college students: Prevalence and correlates from a national survey. Addiction.

[6] Brennan K, Shurmur S, Elhendy A (2004). Coronary artery rupture associated with amphetamine abuse. Cardiology in review.

[7] Pozoga J, Snopek G, Dabrowski M (2005). Acute coronary syndrome after amphetamine use in a young male with myocardial bridging--a case report. Kardiologia polska.

[8] Diercks DB, Fonarow GC, Kirk JD, Jois-Bilowich P, Hollander JE, Weber JE (2008). Illicit stimulant use in a United States heart failure population presenting to the emergency department (from the Acute Decompensated Heart Failure National Registry Emergency Module). The American journal of cardiology.

[9] Cleary M, Jackson D, Woods C, Kornhaber R, Sayers J, Usher K (2017). Experiences of Health Professionals Caring for People Presenting to the Emergency Department After Taking Crystal Methamphetamine (“ICE”). Issues in mental health nursing.

[10] Schürer S, Klingel K, Sandri M, Majunke N, Besler C, Kandolf R (2017). Clinical characteristics, histopathological features, and clinical outcome of methamphetamine-associated cardiomyopathy. JACC: Heart Failure.

